# Supplementary material for: Fitness consequences of depressive symptoms vary between generations: Evidence from a large cohort of women across the 20th century
Source: PLoS One. 2024 Sep 30;19(9):e0310598. doi: 10.1371/journal.pone.0310598 (PMC11441685; doi:10.1371/journal.pone.0310598)
Supplement: S1 Table — Statistics from generalized linear models replacing generation (see Table 2) with age. (DOC) [file pone.0310598.s001.doc]

Supporting information S1

TITLE: Fitness consequences of depressive symptoms vary between generations: Evidence from a large cohort of women across the 20th century

AUTHORS: Christopher I. Gurguis, MD, MS, Renée A. Duckworth, PhD, Nicole M. Bucaro, MD, Consuelo Walss-Bass, PhD

**S1 Table. Generalized Linear Models of Depressive Symptoms and Fitness Components by Age.**

|  | Relative Mating Success | | | Relative Pregnancy Success | | | Relative Birth Success | | |
| --- | --- | --- | --- | --- | --- | --- | --- | --- | --- |
| Variable | d.f. | Chi-Square | p-value | d.f. | Chi-Square | p-value | d.f. | Chi-Square | p-value |
| PHQ-9 | 1 | 66.16 | <0.0001 | 1 | 70.09 | <0.0001 | 1 | 7.96 | 0.0048 |
| Age | 1 | 16.34 | <0.0001 | 1 | 157.08 | <0.0001 | 1 | 150.15 | <0.0001 |
| BMI | 1 | 59.63 | <0.0001 | 1 | 49.47 | <0.0001 | 1 | 37.15 | <0.0001 |
| Race/Ethnicity | 4 | 112.96 | <0.0001 | 4 | 108.41 | <0.0001 | 4 | 112.34 | <0.0001 |
| Level of Education | 4 | 184.38 | <0.0001 | 4 | 166.33 | <0.0001 | 4 | 139.16 | <0.0001 |
| Family Income | 1 | 33.44 | <0.0001 | 1 | 0.08 | 0.7781 | 1 | 0.4 |  |
| PHQ-9 x Age | 1 | 9.83 | 0.0017 |  |  |  |  |  |  |
| Age x BMI | 1 | 35.31 | <0.0001 | 1 | 36.26 | <0.0001 | 1 | 25.91 | <0.0001 |
| Age x Level of Education |  |  |  | 4 | 62.01 | <0.0001 | 4 | 39.49 | <0.0001 |
| BMI x Race/Ethnicity | 4 | 49.2 | <0.0001 |  |  |  |  |  |  |

Statistics from generalized linear models replacing generation (see Table 2) with age.

**Exploration of Non-linear Selection**

Quadratic terms were included in models to explore patterns of non-linear selection on PHQ-9, though we did not include these in the final manuscript. Because proc reg does not allow for interaction terms in SAS, we used proc glm for models including quadratic terms.

Non-linear selection terms were not significant for the regression of quadratic PHQ-9 score on relative mating success in the Silent Generation (F = 2.30, *P* = 0.13), the Baby Boomer Generation (F = 1.98, *P* = 0.16), or Generation X (F = 3.12, *P* = 0.077), but was significant in the Millennial Generation (F = 19.59, *P* < 0.0001). Non-linear selection terms were not significant for the regression of quadratic PHQ-9 score on relative pregnancy success in the Silent Generation (F = 0.00, *P* = 0.95), the Baby Boomer Generation (F = 1.68, *P* = 0.19), Generation X (F = 0.08, *P* = 0.78), or the Millennial Generation (F = 1.77, *P* = 0.076). Non-linear selection terms were not significant for the regression of quadratic PHQ-9 score on relative live birth success in the Silent Generation (F = 0.00, *P* = 0.95), the Baby Boomer Generation (F = 1.18, *P* = 0.28), or Generation X (F = 0.33, *P* = 0.56), but was significant in the Millennial Generation (F = 2.09, *P* = 0.037). In generalized linear models examining the relationship between PHQ-9 score and fitness components, the quadratic PHQ-9 terms was significant for relative mating success (χ^2^ = 14.95, df = 1, *P* < 0.0001), but not for relative pregnancy success (χ^2^ = 0.01, df = 1, *P* = 0.93), or relative live birth success (χ^2^ = 0.99, df = 1, *P* = 0.32)

**Analysis Code**

data evpsych;

input SEQN$ Survey_Year AGE GEN$ GEN_SG GEN_BB GEN_GX GEN_ML BY ETH$ ETH_MXA ETH_NHW ETH_NHB ETH_OTH ETH_OTR EDUC$ EDUC_1 EDUC_2 EDUC_3 EDUC_4 EDUC_5 FMINCBIN$ FMINCBIN2 PHQ9 Number_male_partners PREG_NO BIRTH_NO BMI Missing_all_key_variables$ Missing_analysis_variables$ All_extended_analysis_variables$;

if Missing_analysis_variables =. then delete;

if Missing_all_key_variables =1 then delete;

if All_extended_analysis_variables =. then delete;

if GEN = 'GZ' then delete;

if PREG_NO >15 then delete;

if Number_male_partners >155 then delete;

Rel_MS = Number_male_partners/6.82931206;

Rel_PS = PREG_NO/2.75653041;

Rel_BS = BIRTH_NO/2.05752742;

cards;

/*Data card have been removed from the supporting information. Data used in this analysis may be found in the repository.*/

/*Exploration of data revealed that fitness data and PHQ-9 are right-skewed. Pregnancies >15 and male partners >155 are outliers.*/

proc univariate data=evpsych;

var PHQ9 PREG_NO BIRTH_NO Number_male_partners Age BMI;

histogram PHQ9 PREG_NO BIRTH_NO Number_male_partners Age BMI / normal;

run;

proc sql data=evpsych;

select Number_male_partners, (Number_male_partners - mean(Number_male_partners)) / std(Number_male_partners) as z_scores

from evpsych;

quit;

proc sql data=evpsych;

select PREG_NO, (PREG_NO - mean(PREG_NO)) / std(PREG_NO) as z_scores

from evpsych;

quit;

/*Code for descriptive statistics given in Table 1*/

proc freq data=evpsych;

tables ETH EDUC FMINCBIN;

by GEN;

run;

proc freq data=evpsych;

tables Age*PREG_NO Age*BIRTH_NO Age*Number_male_partners / nopercent norow nocol;

run;

proc anova data=evpsych;

class GEN;

model PHQ9 = GEN;

run;

proc anova data=evpsych;

class GEN;

model AGE = GEN;

run;

proc anova data=evpsych;

class GEN;

model BMI = GEN;

run;

proc anova data=evpsych;

class GEN;

model Number_male_partners = GEN;

run;

proc anova data=evpsych;

class GEN;

model PREG_NO = GEN;

run;

proc anova data=evpsych;

class GEN;

model BIRTH_NO = GEN;

run;

proc freq data=evpsych;

tables ETH*GEN / chisq;

run;

proc freq data=evpsych;

tables FMINCBIN*GEN / chisq;

run;

proc freq data=evpsych;

tables EDUC*GEN / chisq;

run;

/*Code for analyses mentioned in Methods-Generalized linear models*/

proc genmod data=evpsych;

class GEN ETH EDUC FMINCBIN;

model Rel_MS = PHQ9 GEN BMI ETH EDUC FMINCBIN PHQ9*GEN GEN*BMI BMI*ETH / dist=Tweedie link=log type3 wald;

run;

proc genmod data=evpsych;

class GEN ETH EDUC FMINCBIN;

model Rel_MS = PHQ9 GEN BMI ETH EDUC PHQ9*GEN GEN*BMI BMI*ETH / dist=Tweedie link=log type3 wald;

run;

proc genmod data=evpsych;

class GEN ETH EDUC FMINCBIN;

model Rel_PS = PHQ9 GEN BMI ETH EDUC FMINCBIN GEN*BMI GEN*EDUC / dist=Tweedie link=log type3 wald;

run;

proc genmod data=evpsych;

class GEN ETH EDUC FMINCBIN;

model Rel_PS = PHQ9 GEN BMI ETH EDUC GEN*BMI GEN*EDUC / dist=Tweedie link=log type3 wald;

run;

proc genmod data=evpsych;

class GEN ETH EDUC FMINCBIN;

model Rel_BS = PHQ9 GEN BMI ETH EDUC FMINCBIN GEN*BMI GEN*EDUC / dist=Tweedie link=log type3 wald;

run;

proc genmod data=evpsych;

class GEN ETH EDUC FMINCBIN;

model Rel_BS = PHQ9 GEN BMI ETH EDUC GEN*BMI GEN*EDUC / dist=Tweedie link=log type3 wald;

run;

/*Code for analyses mentioned in Methods-Relative fitness components. Proc univariate allows for inspection of data distributions.*/

proc standard data=evpsych mean=0 std=1 out=evpsych_standard;

var PHQ9 Rel_MS Rel_PS Rel_BS;

run;

proc univariate data=evpsych_standard;

var PHQ9 Rel_MS Rel_PS Rel_BS;

histogram PHQ9 Rel_MS Rel_PS Rel_BS / normal;

run;

proc sort data=evpsych_standard;

by GEN;

run;

proc reg data=evpsych_standard;

model Rel_MS = PHQ9;

by GEN;

run;

proc reg data=evpsych_standard;

model Rel_PS = PHQ9;

by GEN;

run;

proc reg data=evpsych_standard;

model Rel_BS = PHQ9;

by GEN;

run;

/*Code for analyses mentioned in supporting information-Exploration of Non-Linear Selection*/

proc glm data=evpsych_standard;

model Rel_MS = PHQ9 PHQ9*PHQ9;

by GEN;

run;

proc glm data=evpsych_standard;

model Rel_PS = PHQ9 PHQ9*PHQ9;

by GEN;

run;

proc glm data=evpsych_standard;

model Rel_BS = PHQ9 PHQ9*PHQ9;

by GEN;

run;

proc genmod data=evpsych;

class GEN ETH EDUC FMINCBIN;

model Rel_MS = PHQ9 PHQ9*PHQ9 GEN BMI ETH EDUC FMINCBIN PHQ9*GEN GEN*BMI BMI*ETH / dist=Tweedie link=log type3 wald;

run;

proc genmod data=evpsych;

class GEN ETH EDUC FMINCBIN;

model Rel_PS = PHQ9 PHQ9*PHQ9 GEN BMI ETH EDUC FMINCBIN GEN*BMI GEN*EDUC / dist=Tweedie link=log type3 wald;

run;

proc genmod data=evpsych;

class GEN ETH EDUC FMINCBIN;

model Rel_BS = PHQ9 PHQ9*PHQ9 GEN BMI ETH EDUC FMINCBIN GEN*BMI GEN*EDUC / dist=Tweedie link=log type3 wald;

run;

/*Code for analyses reported in Tables S1 and S2*/

proc genmod data=evpsych;

class ETH EDUC FMINCBIN;

model Rel_MS = PHQ9 AGE BMI ETH EDUC FMINCBIN PHQ9*AGE AGE*BMI BMI*ETH / dist=Tweedie link=log type3 wald;

run;

proc genmod data=evpsych;

class ETH EDUC FMINCBIN;

model Rel_PS = PHQ9 AGE BMI ETH EDUC FMINCBIN AGE*BMI AGE*EDUC / dist=Tweedie link=log type3 wald;

run;

proc genmod data=evpsych;

class ETH EDUC FMINCBIN;

model Rel_BS = PHQ9 AGE BMI ETH EDUC FMINCBIN AGE*BMI AGE*EDUC / dist=Tweedie link=log type3 wald;

run;

proc genmod data=evpsych;

class ETH EDUC FMINCBIN;

model Rel_MS = PHQ9 BY BMI ETH EDUC FMINCBIN PHQ9*BY BY*BMI BMI*ETH / dist=Tweedie link=log type3 wald;

run;

proc genmod data=evpsych;

class ETH EDUC FMINCBIN;

model Rel_PS = PHQ9 BY BMI ETH EDUC FMINCBIN BY*BMI BY*EDUC / dist=Tweedie link=log type3 wald;

run;

proc genmod data=evpsych;

class ETH EDUC FMINCBIN;

model Rel_BS = PHQ9 BY BMI ETH EDUC FMINCBIN BY*BMI BY*EDUC / dist=Tweedie link=log type3 wald;

run;

/*Code for analyses examining collinearity in our models*/

proc genmod data=evpsych;

model Rel_MS = PHQ9 GEN_SG GEN_BB GEN_GX GEN_ML BMI ETH_MXA ETH_NHW ETH_NHB ETH_OTH ETH_OTR EDUC_1 EDUC_2 EDUC_3 EDUC_4 EDUC_5 FMINCBIN2 / dist=Tweedie link=log type3 wald scoring=50 corrb;

output out=out_ms hesswgt=w;

run;

ods select CollinDiag CollinDiagNoInt;

proc reg data=out_ms;

weight w;

model Rel_MS = PHQ9 GEN_SG GEN_BB GEN_GX GEN_ML BMI ETH_MXA ETH_NHW ETH_NHB ETH_OTH ETH_OTR EDUC_1 EDUC_2 EDUC_3 EDUC_4 EDUC_5 FMINCBIN2 / collin collinoint;

run;

proc genmod data=evpsych;

class GEN ETH EDUC FMINCBIN;

model Rel_PS = PHQ9 GEN_SG GEN_BB GEN_GX GEN_ML BMI ETH_MXA ETH_NHW ETH_NHB ETH_OTH ETH_OTR EDUC_1 EDUC_2 EDUC_3 EDUC_4 EDUC_5 FMINCBIN2 / dist=Tweedie link=log type3 wald scoring=50 corrb;

output out=out_ps hesswgt=w;

run;

ods select CollinDiag CollinDiagNoInt;

proc reg data=out_ps;

weight w;

model Rel_PS = PHQ9 GEN_SG GEN_BB GEN_GX GEN_ML BMI ETH_MXA ETH_NHW ETH_NHB ETH_OTH ETH_OTR EDUC_1 EDUC_2 EDUC_3 EDUC_4 EDUC_5 FMINCBIN2 / collin collinoint;

run;

proc genmod data=evpsych;

class GEN ETH EDUC FMINCBIN;

model Rel_BS = PHQ9 GEN_SG GEN_BB GEN_GX GEN_ML BMI ETH_MXA ETH_NHW ETH_NHB ETH_OTH ETH_OTR EDUC_1 EDUC_2 EDUC_3 EDUC_4 EDUC_5 FMINCBIN2 / dist=Tweedie link=log type3 wald scoring=50 corrb;

output out=out_bs hesswgt=w;

run;

ods select CollinDiag CollinDiagNoInt;

proc reg data=out_bs;

weight w;

model Rel_BS = PHQ9 GEN_SG GEN_BB GEN_GX GEN_ML BMI ETH_MXA ETH_NHW ETH_NHB ETH_OTH ETH_OTR EDUC_1 EDUC_2 EDUC_3 EDUC_4 EDUC_5 FMINCBIN2 / collin collinoint;

run;

quit;
