# Supplementary material for: Fitness consequences of depressive symptoms vary between generations: Evidence from a large cohort of women across the 20th century
Source: PLoS One. 2024 Sep 30;19(9):e0310598. doi: 10.1371/journal.pone.0310598 (PMC11441685; doi:10.1371/journal.pone.0310598)
Supplement: S2 Table — Statistics from generalized linear models replacing generation (see Table 2) with birth year. (DOC) [file pone.0310598.s002.doc]

Supporting information S2

TITLE: Fitness consequences of depressive symptoms vary between generations: Evidence from a large cohort of women across the 20th century

AUTHORS: Christopher I. Gurguis, MD, MS, Renée A. Duckworth, PhD, Nicole M. Bucaro, MD, Consuelo Walss-Bass, PhD

**S2 Table. Generalized Linear Models of Depressive Symptoms and Fitness Components by Birth Year.**

|  | Relative Mating Success | | | Relative Pregnancy Success | | | Relative Birth Success | | |
| --- | --- | --- | --- | --- | --- | --- | --- | --- | --- |
| Variable | d.f. | Chi-Square | p-value | d.f. | Chi-Square | p-value | d.f. | Chi-Square | p-value |
| PHQ-9 | 1 | 27.6 | <0.0001 | 1 | 69.33 | <0.0001 | 1 | 7.6 | 0.0058 |
| Birth Year | 1 | 5.83 | 0.0158 | 1 | 61.32 | <0.0001 | 1 | 171.25 | <0.0001 |
| BMI | 1 | 14.85 | 0.0001 | 1 | 1.85 | 0.174 | 1 | 24.11 | <0.0001 |
| Race/Ethnicity | 4 | 110.98 | <0.0001 | 4 | 105.13 | <0.0001 | 4 | 106.46 | <0.0001 |
| Level of Education | 4 | 179.16 | <0.0001 | 4 | 37.55 | <0.0001 | 4 | 32.72 | <0.0001 |
| Family Income | 1 | 33.71 | <0.0001 | 1 | 0.04 | 0.8512 | 1 | 0.24 | 0.6208 |
| PHQ-9 x Birth Year | 1 | 28.88 | <0.0001 |  |  |  |  |  |  |
| Birth Year x BMI | 1 | 15.23 | <0.0001 | 1 | 1.94 | 0.1631 | 1 | 24.45 | <0.0001 |
| Birth Year x Level of Education |  |  |  | 4 | 38.28 | <0.0001 | 4 | 33.9 | <0.0001 |
| BMI x Race/Ethnicity | 4 | 46.88 | <0.0001 |  |  |  |  |  |  |

Statistics from generalized linear models replacing generation (see Table 2) with birth year.
