# Supplementary material for: Fitness consequences of depressive symptoms vary between generations: Evidence from a large cohort of women across the 20th century
Source: PLoS One. 2024 Sep 30;19(9):e0310598. doi: 10.1371/journal.pone.0310598 (PMC11441685; doi:10.1371/journal.pone.0310598)
Supplement: S3 Table — Statistics from generalize linear models removing family income due to detected collinearity (compare with Table 2). (DOC) [file pone.0310598.s003.doc]

Supporting information S3

TITLE: Fitness consequences of depressive symptoms vary between generations: Evidence from a large cohort of women across the 20th century

AUTHORS: Christopher I. Gurguis, MD, MS, Renée A. Duckworth, PhD, Nicole M. Bucaro, MD, Consuelo Walss-Bass, PhD

**Assessing for Collinearity in Generalized Linear Models**

Collinearity among weighted predictor variables was assessed by first creating dummy variables for all categorical variables, then fitting new models using Fisher’s scoring and the hesswgt= option in proc genmod. The diagonals of the generated weight matrix were then used in a weighted regression using proc reg. This procedure is recommended to evaluate for collinearity in proc genmod by SAS (<https://support.sas.com/kb/32/471.html>) since VIF cannot be obtained in proc genmod*.* The condition index was low for all variables except the highest two levels of education and family income, indicating high collinearity between these. When we repeat analyses after removing family income from the models, patterns of results remain the same as those presented in the main manuscript (compare S3 Table with Table 2). We therefore elected to retain family income in our final models due to the known unique, if interrelated, impact of socioeconomic status and education independently on mental illness in the literature.

**S3 Table. Generalized Linear Models of Depressive Symptoms and Fitness Components with Family Income Covariate Removed**

|  | Relative Mating Success (β=0.044) | | | Relative Pregnancy Success (β=0.011) | | | Relative Birth Success (β=0.0039) | | |
| --- | --- | --- | --- | --- | --- | --- | --- | --- | --- |
| Variable | d.f. | Chi-Square | p-value | d.f. | Chi-Square | p-value | d.f. | Chi-Square | p-value |
| PHQ-9 | 1 | 196.31 | <0.0001 | 1 | 66.75 | <0.0001 | 1 | 6.14 | 0.0132 |
| Generation | 3 | 19.50 | 0.0002 | 3 | 190.82 | <0.0001 | 3 | 166.82 | <0.0001 |
| BMI | 1 | 6.80 | 0.0091 | 1 | 28.01 | <0.0001 | 1 | 31.50 | <0.0001 |
| Race/Ethnicity | 4 | 109.16 | <0.0001 | 4 | 111.64 | <0.0001 | 4 | 106.19 | <0.0001 |
| Level of Education | 4 | 163.46 | <0.0001 | 4 | 550.45 | <0.0001 | 4 | 543.13 | <0.0001 |
| PHQ-9 x Generation | 3 | 22.20 | <0.0001 |  |  |  |  |  |  |
| Generation x BMI | 3 | 30.38 | <0.0001 | 3 | 39.15 | <0.0001 | 3 | 33.33 | <0.0001 |
| Generation x Level of Education |  |  |  | 12 | 209.67 | <0.0001 | 12 | 131.06 | <0.0001 |
| BMI x Race/Ethnicity | 4 | 46.36 | <0.0001 |  |  |  |  |  |  |

Results of generalized linear models of the relationship between each fitness component and PHQ-9 corrected for important covariates and the interactions between these covariates. β indicates the strength of the relationship for each component.
